# Butterfly Girls; promoting healthy diet and physical activity to young African American girls online: rationale and design

**DOI:** 10.1186/1471-2458-13-709

**Published:** 2013-08-02

**Authors:** Debbe Thompson, Rory Mahabir, Riddhi Bhatt, Cynthia Boutte, Dora Cantu, Isabel Vazquez, Chishinga Callender, Karen Cullen, Tom Baranowski, Yan Liu, Celeste Walker, Richard Buday

**Affiliations:** 1 USDA/ARS Children’s Nutrition Research Center, Baylor College of Medicine, 1100 Bates Street, Houston TX 77030, USA; 2 Department of Family and Community Medicine, Baylor College of Medicine, 3701 Kirby Dr., Suite 600, Houston TX 77098, USA; 3 Playwright, Houston, TX, USA; 4 Archimage, 4203 Montrose Boulevard., Suite 390, Houston, TX 77004, USA

**Keywords:** Internet, Intervention, Obesity prevention, Physical activity, Child, African American, Diet, Culture

## Abstract

**Background:**

Young African American girls have a high risk of obesity. Online behavior change programs promoting healthy diet and physical activity are convenient and may be effective for reducing disparities related to obesity. This report presents the protocol guiding the design and evaluation of a culturally and developmental appropriate online obesity prevention program for young African American girls.

**Methods/Design:**

*The Butterfly Girls and the Quest for Founder’s Rock* is an 8-episode online program delivered as an animated, interactive comic. The program promotes healthy diet and physical activity and is specifically designed for 8–10 year old African American girls. Girls, parents, and community representatives provided formative feedback on cultural relevance and developmental appropriateness. A three-group (treatment, comparison, wait-list control) randomized design (n = 390 parent/child dyads) is employed, with child as the unit of assignment. Change in body mass index is the primary outcome; change in fruit and vegetable consumption, water, and physical activity are secondary outcomes. Data collection occurs at baseline, approximately 3 months after baseline (i.e., completion of the online program), and approximately three months later (i.e., maintenance assessment). Two dietary recalls are collected at each data collection period by trained interviewers using the Nutrient Data System for Research (NDSR 2012) system. Physical activity is objectively measured by seven days of accelerometry. Psychosocial and process data are also collected. Girls in the treatment and comparison groups will be interviewed at post 1 to obtain information on personal reactions to the program.

**Discussion:**

This research will develop and evaluate the efficacy of an online program for reducing obesity risk among girls at risk of obesity and related diseases. Online programs offer the potential for wide dissemination, thus reducing disparities related to obesity.

**Trial Registration:**

NCT01481948

## Background

Ethnic minorities in the United States have higher burden of chronic disease and death than non-minorities [1]. Because obesity increases chronic disease risks [2], obesity prevention among ethnic minorities is a national priority [3] that has the potential to decrease obesity-related health disparities. Since obesity, particularly during adolescence, tracks into adulthood [4], intervening among at-risk groups prior to this critical period is essential. African American girls [5] have a high prevalence of obesity and their diet and physical activity (PA) choices place them at increased risk [6-8]. Interventions targeting healthful change in African American girls’ diets and PA behaviors prior to adolescence may reduce immediate and future obesity risks by establishing skills and behaviors that enable them to achieve and maintain a healthy body weight.

Interventions must reflect the realities and expectations of the intended audience [9]. Programs designed for ethnic minorities should embody cultural awareness and sensitivity [9] at both surface (i.e., appropriate language and role models) and deeper levels (e.g., knowledge of cultural behaviors, beliefs regarding the targeted behavior(s), and awareness of broadly shared cultural values and expectations) [10]. Interventions developed for children and adolescents must also be developmentally appropriate (i.e., expectations, concepts, and skills should be presented in a way that a child comprehends and is able to perform) to facilitate acquisition of essential knowledge and skills [11]. Activities may facilitate the transfer of knowledge and skills from the learning environment (i.e., online program) to the “real-world” through active learning – i.e., learning environments where participants are actively involved in the learning process [12]. One way to achieve this may be through an interactive story-based environment [13] (such as an online comic) that has appealing characters [14]. Since observational learning is one of the most common ways in which people learn [15], embedding key behavior change procedures (e.g., goal setting, self monitoring, and problem solving) [16,17] in an engaging story where characters model these procedures to overcome problems may be an effective method for promoting behavior change among youth [13]. This may be particularly effective when the story is immersive, and the characters are viewed as similar [18].

Lack of access to preventive programs has contributed to health disparities [19,20]. Using the internet to deliver obesity prevention programs offers the potential to increase access. Although there have been concerns that the internet would enhance health disparities [21], internet access among people of all income groups, races, and ethnicities has increased [22,23]. Because internet use among youth is high [24], online programs offer an opportunity to integrate key behavior change components into their design [25,26]. Online programs enable materials to be delivered in the same way to each participant without the potential contamination that may be introduced through live instructors [27]. However, to be effective, programs need to be vetted with key stakeholders to ensure cultural relevance, comprehension, and appeal [26,28].

Although developmentally appropriate and culturally relevant online behavior change programs hold promise as an effective method for reaching youth [29-34], few programs promoting healthy diet and/or physical activity have been specifically designed for pre-adolescent African American girls [7,35-39]. Further, little research has been conducted to examine the additive effects of behavior change procedures such as goal setting, self monitoring, and goal reporting on behavioral and psychosocial outcomes. This report describes the protocol guiding the design and evaluation of *The Butterfly Girls and The Quest for Founder’s Rock* (BFG), an online obesity prevention program specifically designed for 8–10 year old African American girls.

## Methods/Design

### Study design

This evaluation employs a three-group, randomized control design, with three data collection periods: baseline, post 1 (immediate post program completion), post 2 (3 months later). Following baseline assessment, girls are randomized to one of three groups (treatment, comparison, wait list control). Girls in the treatment group engage in theory-prescribed behavior change procedures (e.g., goal setting, goal reporting, self monitoring, feedback on goal attainment) embedded in online, interactive, role modeling comics. The comparison group receives the online comics without the theory-prescribed behavior change procedures. After participating in all three data collection periods (i.e., approximately six months after completing baseline assessment), girls in the wait-list control group receive the treatment intervention. A wait-list group controls for potential threats to internal validity, such as history and maturation.

**Figure 1:**
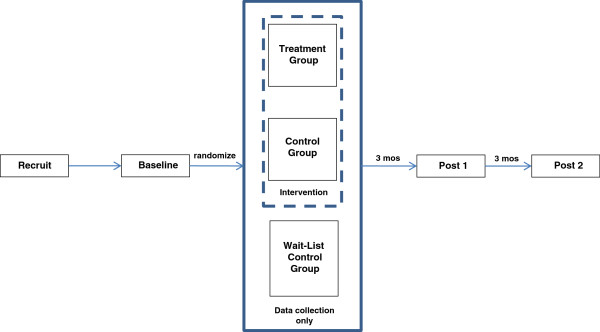
Research design.

Girls have up to three months to view all 8 episodes. For girls in the treatment and comparison conditions, post 1 data collection occurs immediately upon completion of the 8 episodes or approximately 3 months after playing episode 1, whichever occurs first; post 2 data collection occurs three months after post 1 (Figure 1). The protocol was approved by the institutional review board at Baylor College of Medicine (H-27505) and registered with ClinicalTrials.gov (NCT01481948).

### Participants

Eligibility criteria are: an 8 to 10 year-old African American girl with a personal email address, internet access, and a parent or legal guardian who allows their child to participate and is willing to participate in the parent component. Recruitment techniques utilize standard procedures (i.e., flyers, attendance at community events), the volunteer database at the Children’s Nutrition Research Center (CNRC), and a video trailer posted to the CNRC volunteer website and newsletter. Prior to participation, written informed consent and assent is required. Child is the unit of randomization and assignment. Recruitment began in November 2012. The outcome study began enrolling participants in January 2013.

### Sample size and power

The primary outcome is body mass index (BMI) percentile. Therefore, estimated sample size (SS) was based on the number of participants needed to detect a significant intervention group by time interaction effect for BMI percentile. Data from the Baylor GEMS study of 8–10 year old African American females yielded a baseline BMI percentile of 89.4 ± 14.7 (n = 35) [35]. Given a 0.05 level of significance, a pooled standard deviation of 14.7, and autocorrelations 0.50, a sample of 324 participants would be adequately (≥80%) powered to detect a small (SEF; standardized effect size, f = 0.13) group by time interaction. This sample size would detect a 4.1% increase in BMI percentile across time in the comparison and wait list control groups while the treatment group BMI percentile remains stable. Given a final sample of 324 participants and allowing for a 20% attrition rate, 390 participants will be recruited.

### Setting

Parents and children participate in separate intervention and data collection activities electronically (online, telephone) from locations of their choice (home, community).

### Child intervention

#### Overview

Children view an eight-episode online program that promotes five servings of fruit and vegetables (FV), five glasses of water (40 fl oz), and 60 minutes of PA per day. Girls in the treatment group set and report goals, self monitor, and receive feedback related to level of goal attainment while comparison group girls do not engage in these activities.

#### Development

The original online program was developed as part of the Girls Health Enrichment Multi-site (GEMS) Program initiative funded by the National Heart, Lung, and Blood Institute, National Institutes of Health, to identify ways to reduce obesity risk among 8–10 year old African American girls [40]. The 12-week Baylor College of Medicine-GEMS project (*Fun, Food, & Fitness*), guided by Social Cognitive Theory [15] and Elaboration Likelihood Model [41,42], consisted of a 4-week summer day camp, followed by an eight-week internet intervention [35,42]. The online program included role-modeling comics where six African American girl characters modeled key skills targeted by the intervention program, including problem solving, decision making, asking/negotiation, and goal setting to meet FV, water, and PA goals: each day girls attempted to consume five servings of FV, ≥five 8 fl oz glasses of water, and attain 12,000 steps. After viewing role-modeling comic sequences, the girls participated in personal goal setting using simple schemas and goal reporting components. Girls were asked to report goal attainment for the previous day only. Self-monitoring was encouraged. After completing the required activities (comics plus goal setting and goal review), the girls had access to “fun pages” that provided information of interest to the girls, such as brief profiles of the comic characters and homework help. The program had limited interactivity and animation [42].

The pilot test of the Baylor College of Medicine-GEMS project (n = 35) demonstrated high camp attendance in the treatment group, but low login rates to the online program [35]. Although not statistically significant due to the small sample size, diet and PA changes were in the desired directions [35]. A subsequent pilot study tested the online program as a stand-alone intervention. Renamed *Food, Fun, & Fitness Internet Program for Girls,* a brief story introduction was added to provide context (i.e., reasons the girls formed a club and were trying to make healthy diet and PA choices) (n = 78)*.* High logon rates [43] and statistically significant change in self-reported FV intake and PA were observed [30]. Media advertisements resulted in numerous calls to inquire about the study [44], suggesting community interest. Because these results were promising, there was a need to conduct an efficacy study with a larger, more adequately powered sample and stronger measures of diet and PA to examine short and longer term effects on obesity risk.

Although steps were taken to ensure cultural appropriateness and sensitivity in the original program [45], it was developed [46] and tested [35] over a decade ago. Therefore, to ensure appeal, cultural relevance, and developmental appropriateness with key stakeholders (i.e., girls, parents, and the community), formative work was conducted prior to the efficacy study to identify needed modifications.

##### Interviews/surveys

Three expert panels reviewed program components to ensure appeal and cultural and developmental appropriateness: girls (n = 20), parents (n = 20), and community representatives (n = 10). Girls and parents participated in interviews, and the community panel completed online surveys. Similar questions were asked of parents, girls, and community representatives. Interviews were conducted by trained staff following a semi-structured script; probes and prompts were used to expand and clarify responses. Interviews were digitally recorded and lasted approximately 1.5 hours each. Surveys were completed over a secure, password protected website. Stipends were provided for each interview ($40 girl, $40 parent) and survey ($50) completed.

The first round of data collection reviewed the existing program to identify needed changes to structure and content. Modifications made as a result of the first round of data collection included changing the characters (e.g., more diversity in facial features, body sizes, skin tones, hair styles; updated clothing; more contemporary names) and enhancing the behavioral components (e.g., reporting goal attainment for 7 days vs 1 day each week; providing positive feedback statements on goal attainment). A richer story was added, interactivity was enhanced, graphics were updated, sound effects were enriched, and a new vocal track was created. A second round of data collection was conducted to review the modifications. Although several minor modifications to characters’ appearances were suggested, the overall program met with the approval of parents, girls, and community representatives. The program is described below.

##### Theoretical framework

The theoretical framework guiding the BFG program [42] is comprised of Social Cognitive Theory [15] and the Elaboration Likelihood Model [41]. Social Cognitive Theory provided support for observational learning, and the self-regulatory behavior change procedures (e.g., goal setting, self monitoring, problem solving, feedback) to promote personal mastery. Elaboration Likelihood Model provided guidance on character development.

##### Story

The original program did not have an overall storyline. Instead, online comics were vignettes loosely connected by setting and characters. To promote immersion and enhance appeal [14], a storyline was developed that unfolds in eight episodes. In the original program [42], goal setting and reporting were separate from the comics. In contrast, BFG integrates the behavior change components into the story. In keeping with the theme of the story and to enhance appeal, the name of the updated online program was modified to *The Butterfly Girls and the Quest for Founder’s Rock.*

##### BFG Storyline

Girlfriends from MacGuffin Middle School are teased at camp by boys as they watch the girls take pictures of butterflies. As a result, the boys mockingly call the girls "butterflies." Back in school, the girls discover the boys have entered a citywide competition to find the legendary Founder’s Rock, which is the place where their town of MacGuffin Springs was first settled. The girls think the competition is a perfect opportunity to show the boys “who is superior to whom.” The girls decide to form The Butterfly Club and enter the competition. Each team has to run all over the city looking for clues. The first team to find Founder's Rock wins. Although the boys are sure the girls can't keep up, the girls discover that eating FV, drinking water, and being physically active help them gain the stamina needed to find the clues to win the contest. Despite seemingly insurmountable obstacles, The Butterfly Girls win the competition, eventually sharing their “secret” advantage (healthy diet and PA) with the boys.

##### Characters

**Figure 2:**
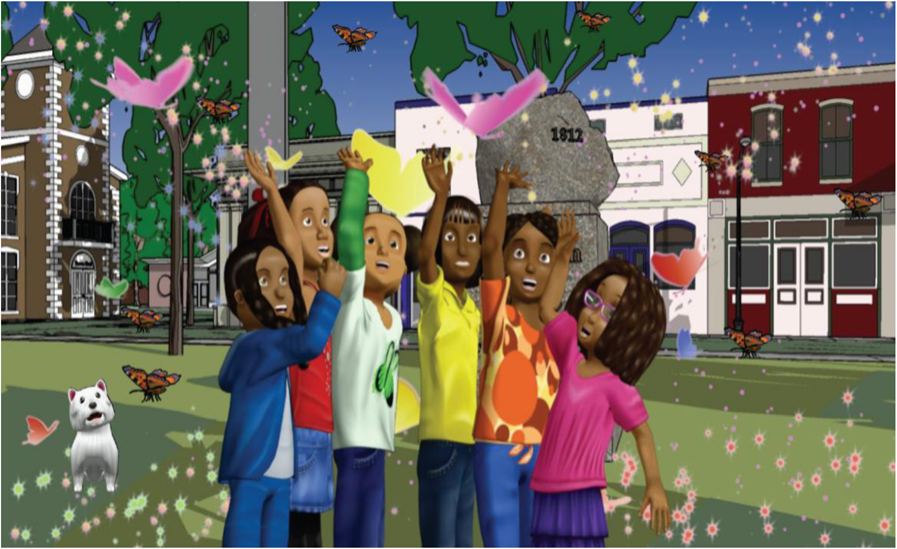
Characters.

The protagonists (non-player characters) are six young African American girls approximately 8–10 years of age (Figure 2) and the Player (i.e., the study participant). Each character has a different look, personality, and specific struggle related to the targeted behaviors. The characters serve as role models by demonstrating key behavior change strategies and a coping style where they struggle to overcome problems related to goal attainment [47]. Antagonists are African American boys of a similar age to the girls who are the star players on the MacGuffin Middle School soccer team.

##### Program structure

BFG is a browser-based interactive comic developed in HTML5 using Hype 1.5 (Tumult Inc, San Francisco, CA, 2012). The comics include voice-over narration performed by professional voice talent, a custom music score (R. Buday, composer) and sound effects. Characters and settings were created as three-dimensional models and rendered as still images. The story genre is action adventure told from a second person perspective. Participants in the treatment group engage in activities designed to promote personal mastery (e.g., goal setting, goal review, self monitoring, tailored feedback), while those in the comparison group do not engage in these activities [15].

**Figure 3:**
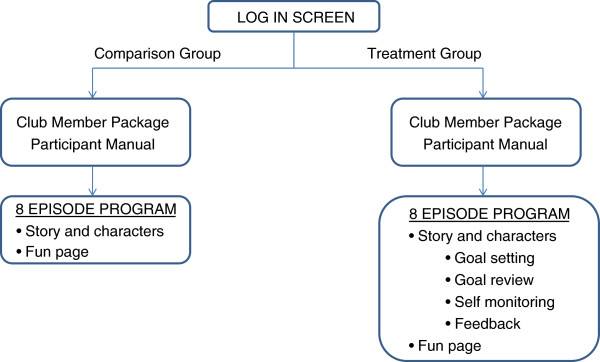
Flow diagram.

The story unfolds in eight episodes. Art assets were created by a professional artist. The participant is represented in the story by an unseen character referred to as “Rookie,” since she is the newest member of the Butterfly Girls Club. Rookie has a dog named, "Wok" who makes humorous observations conveyed as thought bubbles. Character comments are voiced and also appear as on-screen text. Interactivity is included to help participants feel part of the story. For example, participants are encouraged to “explore” comic panel scenes to enhance immersion and enjoyment. At certain times, clicking on a character reveals their interior thoughts (i.e., private thoughts not voiced or critical to the behavior change components). Rolling over objects in a scene also elicits tooltips (e.g., “This is a blender”). A rich audio track with music and sound effects is included. The program structure is presented in Figure 3.

##### Beta testing

Multiple rounds of internal testing were conducted to identify technical issues, discrepancies between storyboards and the programmed episodes, and other issues that needed to be addressed prior to completing the program. This testing revealed that an Apple Safari or Google Chrome browser, Apple QuickTime, and Adobe Acrobat Reader (or equivalent PDF reader) software were needed by participants. Links and directions regarding how to download and install these programs are available.

##### Login screen

**Figure 4:**
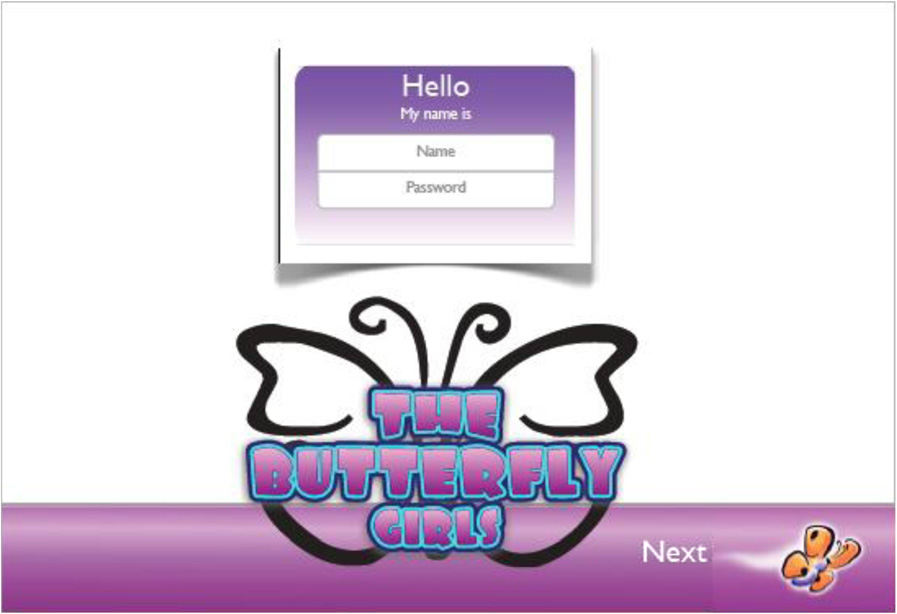
Log in screen.

Participants receive a unique username and password to logon to the online program. This information automatically routes them to their assigned version of the program (treatment, comparison) (Figure 4).

##### Goal setting

Girls in the treatment group set goals in each episode. To facilitate goal setting and attainment, girls are presented with schemas during goal setting. The schemas have been described elsewhere [42]. Schemas were developed for each behavior promoted in the study (FV, water, PA). For example, four schemas were created for FV. The schemas vary by when the participant eats FV each day (i.e., meal, snack): a Breakfast Builder eats two servings of FV at breakfast and one each at lunch, dinner, and snack, while a Dynamite Diner eats two FV servings at dinner and one each for other meals and snack. Similar schemas were developed for water and PA.

**Figure 5:**
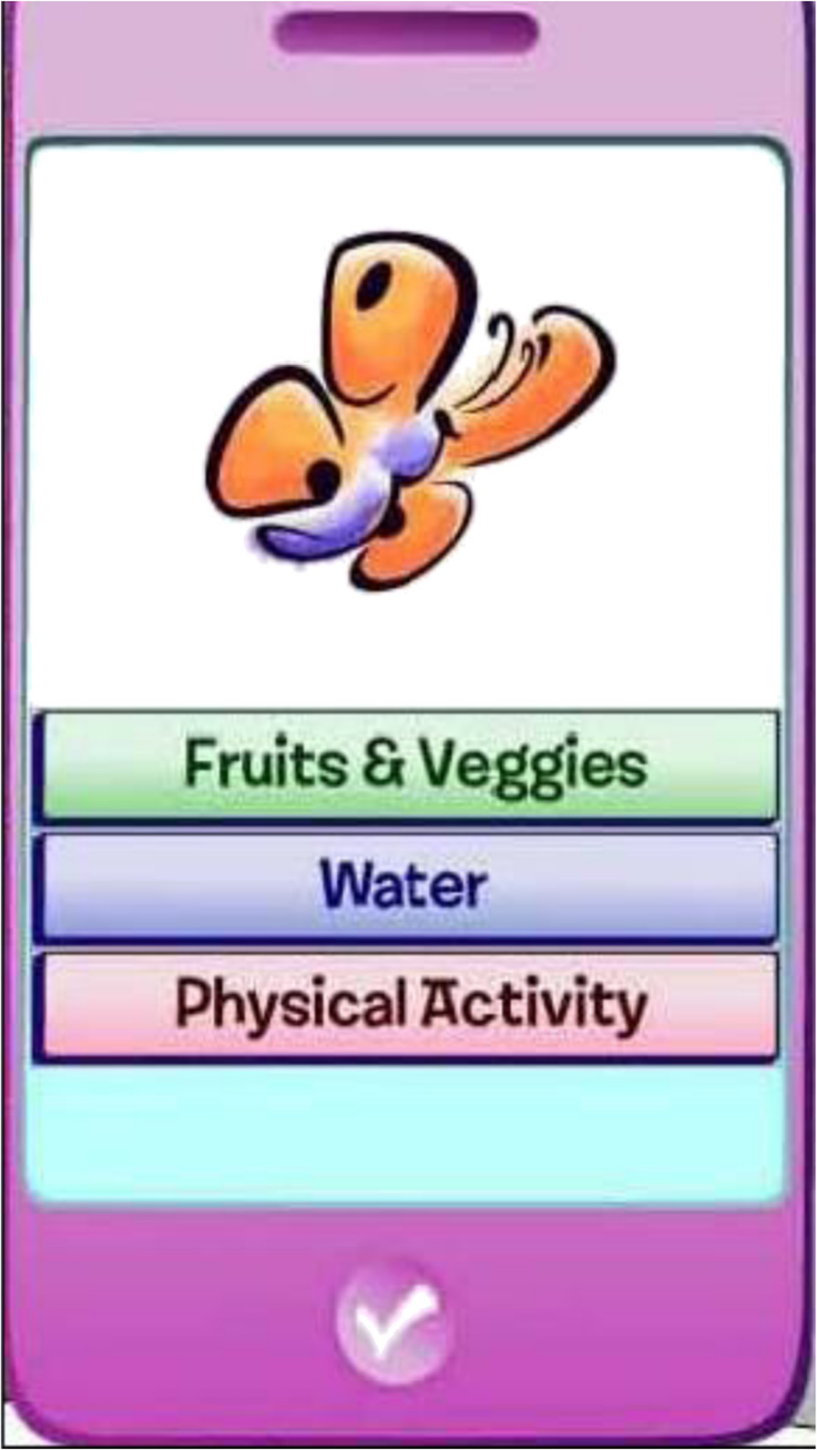
Goal setting.

**Figure 6:**
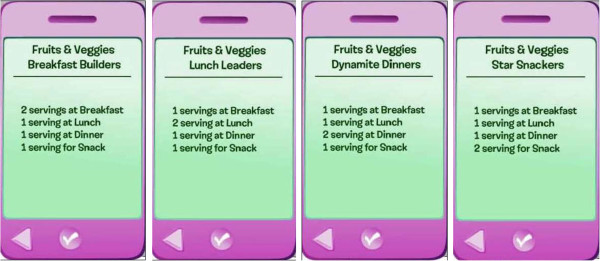
FV schemas.

Characters and the study participant use a virtual "iPod" to set goals during the online program. Figure 5 is a screenshot of the way goal setting is presented in the program. When each behavior is clicked on, a page with the schemas for that behavior opens. Girls click on a schema to select it (Figure 6 portrays the FV schemas in the virtual "iPod" format).

##### Self monitoring

**Figure 7:**
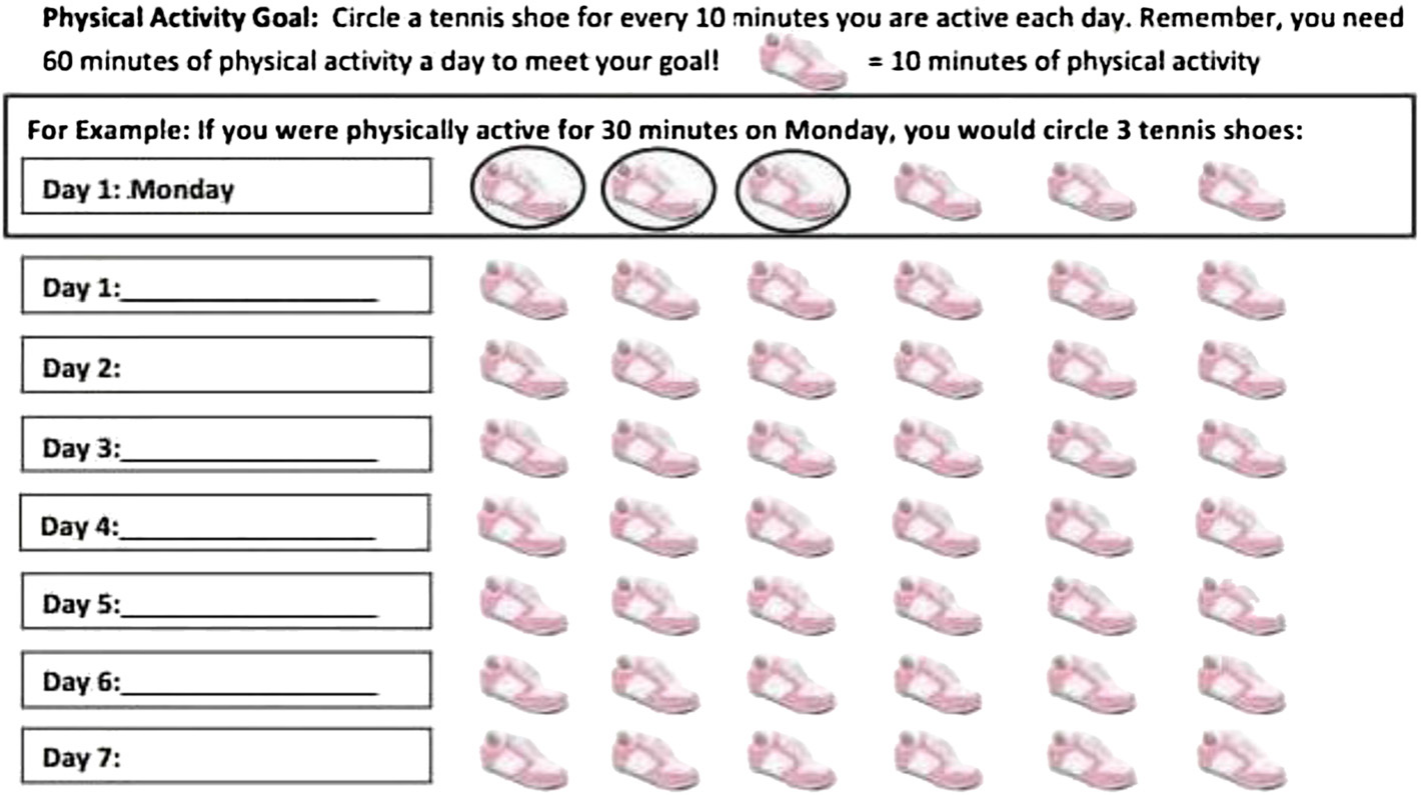
PA tracking sheet.

Girls in the treatment group are asked to keep track of progress towards their goals. The tracking form is presented in Figure 7. The girls use this to help them complete goal review each episode.

##### Goal review

**Figure 8:**
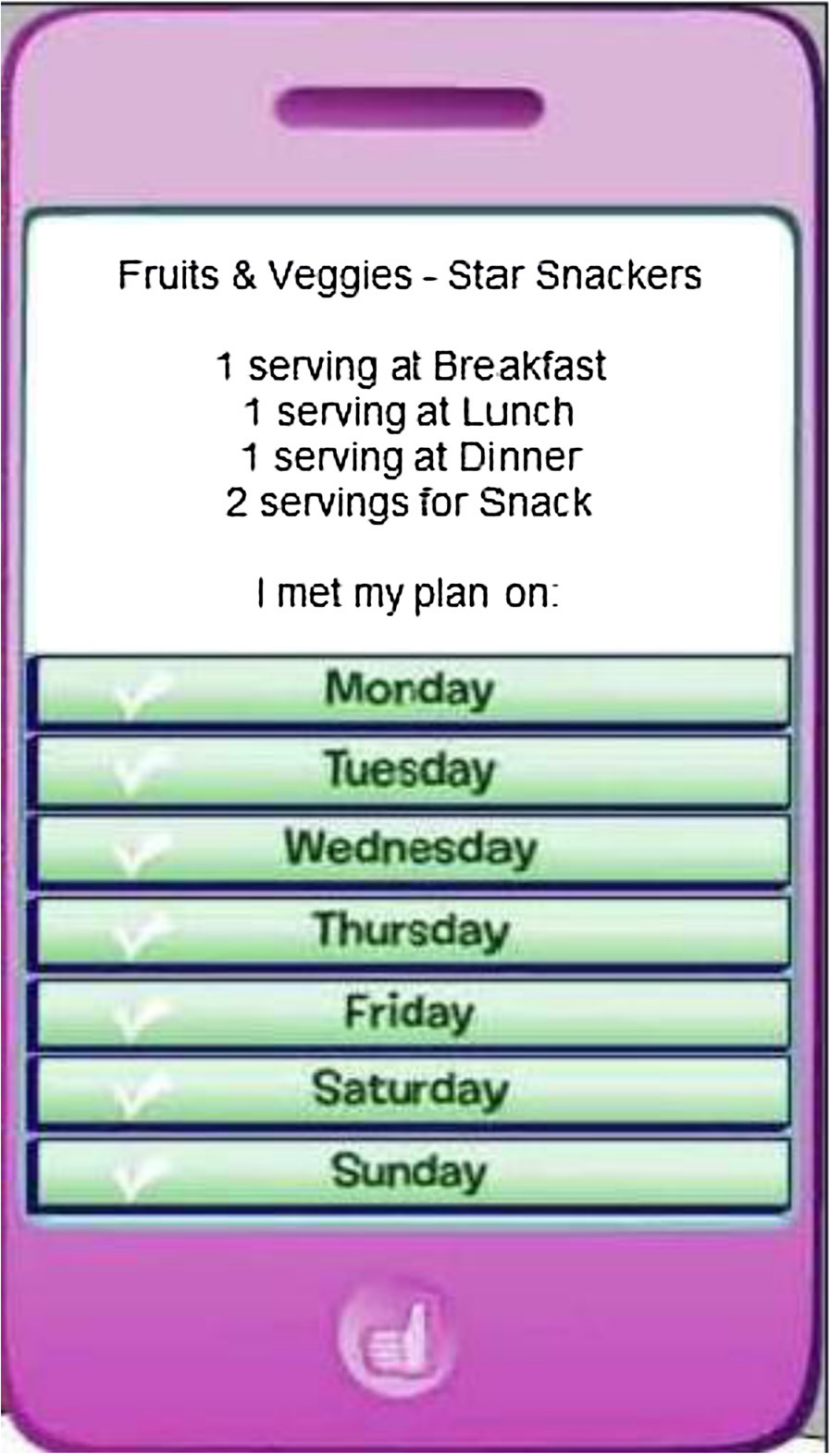
Goal review.

This component appears in episodes 2–8 (treatment group only). Participants report their goal attainment via a “virtual” iPod. The schema they selected the previous episode appears on the computer screen, and girls report the days they met their goal for each behavior. Based on level of goal attainment, girls then receive feedback from one of the characters (Figure 8).

##### Skills

**Figure 9:**
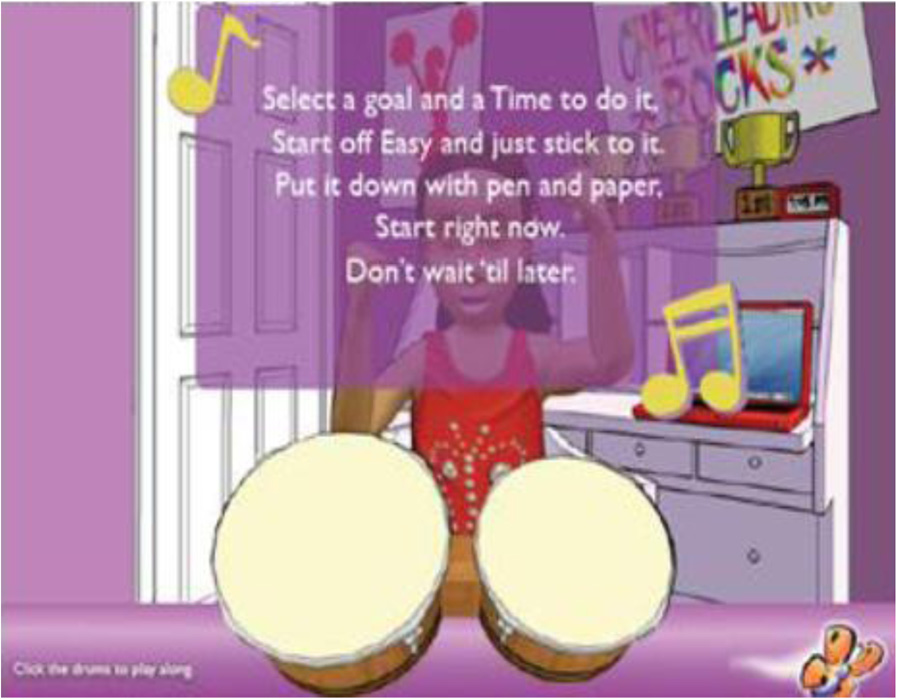
Poem.

As in the original program [42], different problems and skills are emphasized each episode. Updates to this component are presented in (Table 1). One of the characters is a musician and a poet. In the treatment group, when one of the Butterfly Girls faces a problem, this character creates a poem and sets it to music (aka percussive poetry); the poem serves as a heuristic to help the participant remember how to use the skill [42]. The participant can “play along” with the characters when the poem is voiced by clicking on bongos that appear on the screen (Figure 9). In the comparison group, the characters identify the skill used to solve the problem, but do not model how to use it.

**Table 1:** Skill and problem focus by episode

**EPISODE**	**SKILL FOCUS**	**PROBLEM FOCUS**
1	Problem solving / decision making	PA at home
2	Problem solving / decision making	FV for snack
3	Asking/negotiation	FV home availability/accessibility
4	Goal setting	PA with parent
5	Self monitoring	Water vs soda
6	Problem solving /decision making / goal setting	Homework
7	Asking/negotiation	PA
8	Problem solving / decision making	FV when eating out

##### Fun page

**Figure 10:**
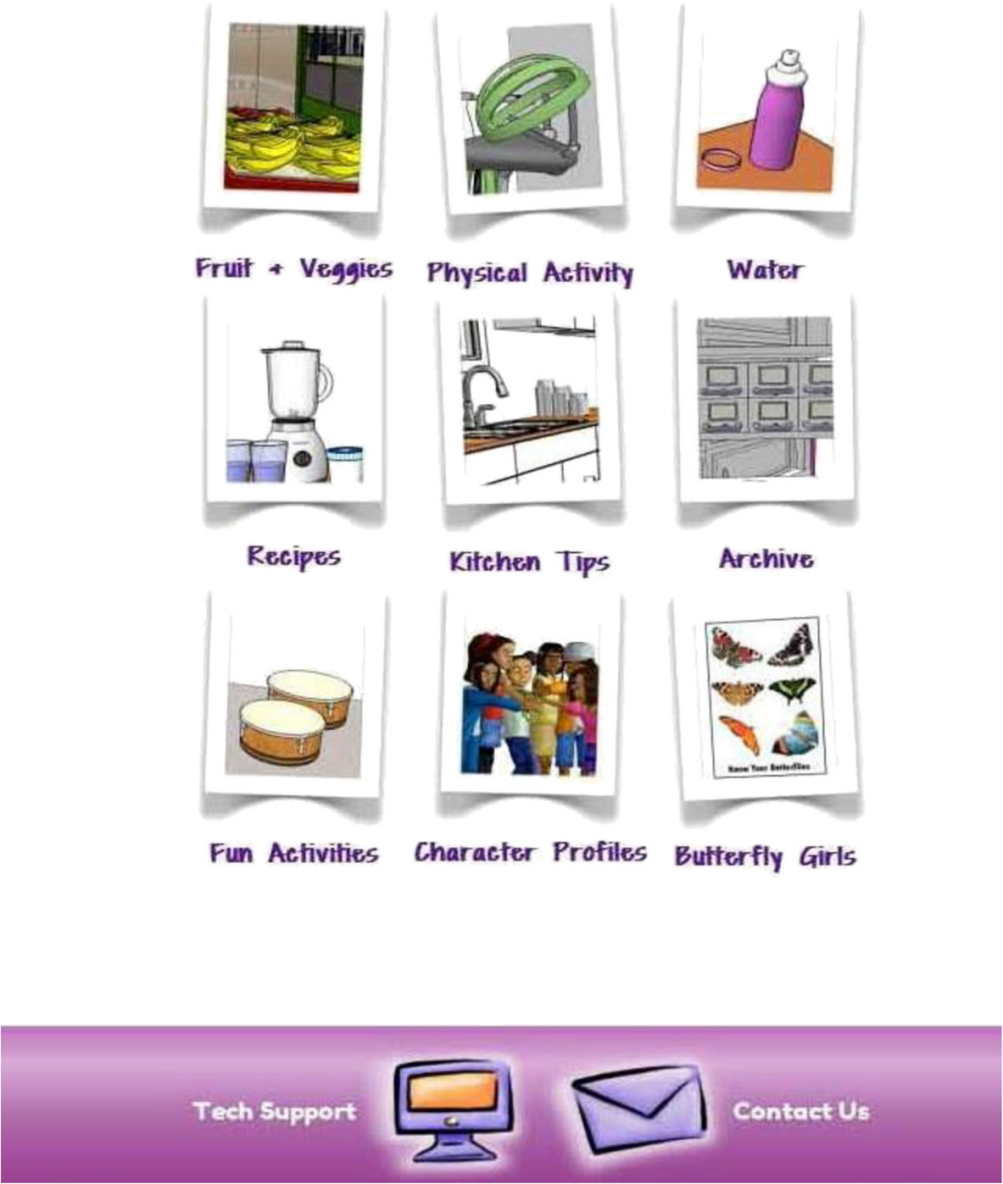
Fun page.

The Fun Page (Figure 10) provides participants with a wide range of information and activities related to diet (e.g., water, FV, portion sizes, making healthy choices at restaurants), PA, kitchen safety, and developmentally-appropriate FV recipes they can prepare with a parent (e.g., trail mix, smoothie). This section also includes fun activities (e.g., crossword puzzles, word finds, matching activities) where participants use FV, water, and PA knowledge taught in the program to complete the activity. Additionally, the Fun Page contains a listing of free local activities (e.g., parks, museums), BFG character profiles, and archived episodes of the BFG previously viewed by the participant.

#### Parent component

**Figure 11:**
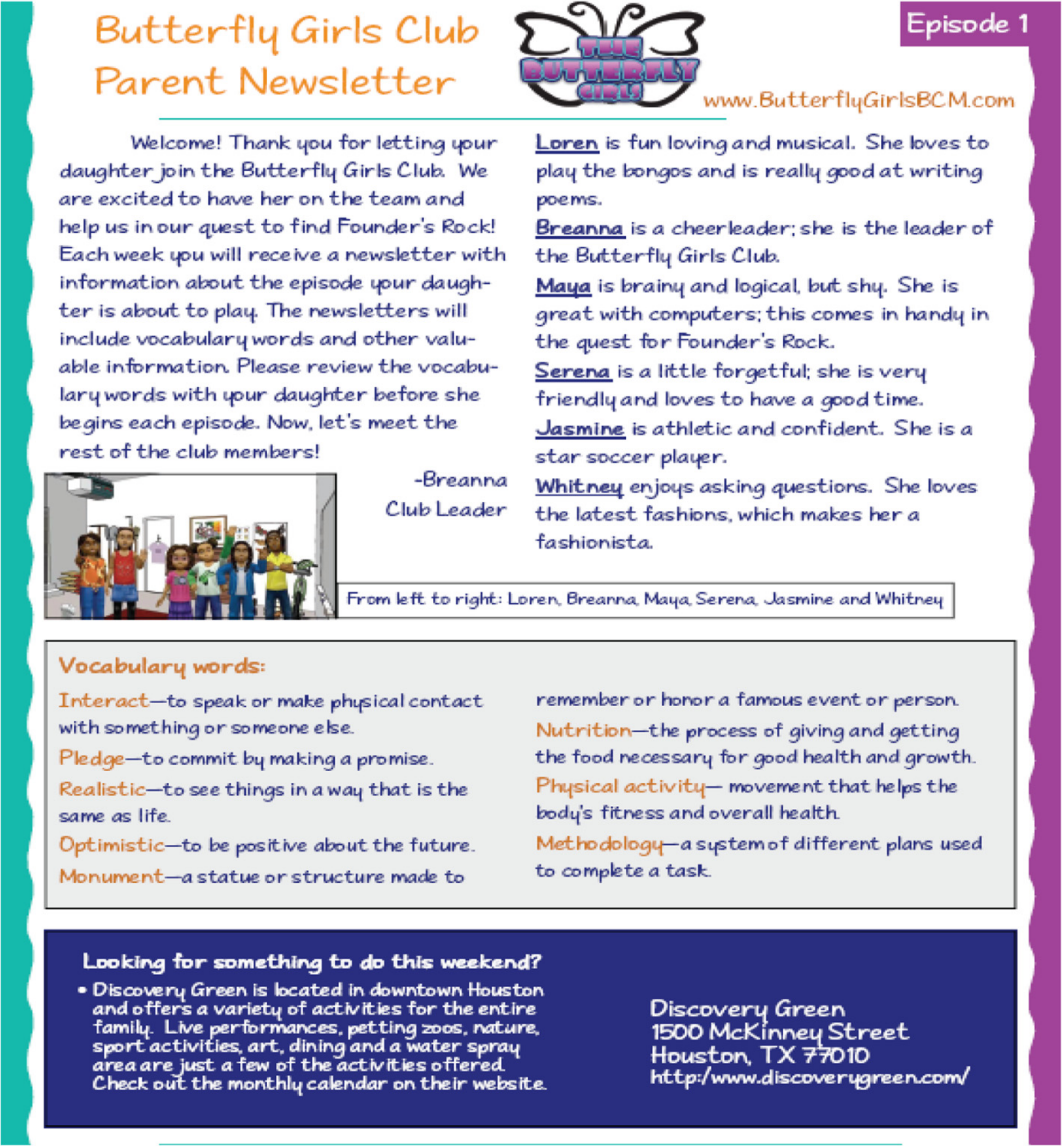
Parent newsletter.

Parents receive electronic newsletters that correspond to each episode of the program the girls receive. The newsletters contain some or all of the following, depending on the episode: vocabulary words for the upcoming episode that parents are asked to review with their daughter prior to playing the next episode, healthy recipes, tips, and fun places to visit in Houston (e.g., museums, parks) (Figure 11). Parents need Adobe Acrobat Reader software (available at no cost) installed on their computer to view the newsletters. A link and directions to download this software are available.

### Procedures

#### Randomization

A block randomization scheme is used to allocate subjects to the three groups (treatment, comparison, wait list control). The block strategy ensures balance in the randomization process such that equal numbers of participants are assigned to each group. The computer algorithm written in SAS® (version 9.3, 2012, Cary, NC) performed the block randomization using the PROC PLAN procedure and the computer system clock for the random seed [48].

#### Intervention

After completing baseline assessment, girls are randomized to condition. After randomization, girls in the treatment and comparison groups receive a “Club Member Package” delivered to them via courier service to welcome them into the study. The package includes a BFG logo t-shirt,a bracelet with “Butterfly Girls” stamped into it, and a 20 fl oz logo water bottle. A bottle of this size was selected because drinking two filled bottles a day will meet their water goal (five 8 fl oz servings). The package also includes a participant manual targeted to their study group. The participant manual for both treatment and comparison participants contains helpful information, such as the backstory (i.e., reason the characters formed the BFG Club), guidelines for navigating the website, and how to resolve technical problems. The manual for the participants randomized to the treatment condition also contains guidelines for how to set and report goals, as well as pre-printed tracking sheets. Girls randomized to the wait-listed control group receive these materials immediately prior to beginning the online program.

Participants are eligible to play one episode each week after they complete the previous episode. This provides them with time to meet the goals they set the previous episode. They receive an automatic email each time an episode becomes available. Simultaneously, the parent receives an email with a link to an episode-specific newsletter. When a participant completes an episode, an email is automatically generated and sent to the research staff. The same procedure occurs when parents open a newsletter. If the participant does not view an episode within three days of eligibility to play the episode, they receive an email reminder. After six days, a reminder call is made to the parent. The parent then receives a call every five days until the child completes the episode (maximum of five calls). At this point, a decision is made as to whether to move the participant to inactive status. The program is structured so that girls will not miss an episode. For example, if they are eligible to play episode 2 but do not logon for three weeks, when they logon, they have access to episode 2.

#### Data Collection

All self-report data are collected over a secure, password protected website. Parents and children are provided with separate passwords. A thorough process evaluation is also being conducted using the framework proposed by Baranowski and Jago [49]. Examples of data collected include staff logs of recruitment, enrollment, participant contacts, and technical issues. Gameplay data (i.e., player input data) are collected as girls navigate the program. These data include logins, goals set, goals attained, and locations visited on the fun page. Data are also collected regarding the number of times parents click on links to the newsletters. During post 1 data collection, parents and girls are asked to self-report program appeal and use, and girls participate in a brief interview to assess program appeal and whether it met their expectations (Table 2).

**Table 2:** Data collection

**WHO**	**WHAT**	**BASELINE**	**DURING**	**POST 1**	**POST 2**
**GIRL**	Height	x		x	x
	Weight	x		x	x
	BMI percentile (calculated)	x		x	x
	Diet (2 recalls (NDSR 2012) [50]	x		x	x
	PA (7 days of accelerometry)* [51]	x		x	x
	FV preferences [52]	x		x	x
	Asking behaviors [53]	x		x	x
	Self efficacy [54]	x		x	x
	Outcome expectancies [55]	x		x	x
	Immersion [26]			x	
	Program appeal/use [26]			x	
	Social Desirability [56,57]	x			
	Interview [26]			x	
	Game-play [26]		x		
**PARENT**	FV intake [58]	x		x	x
	FV home availability [59]	x		x	x
	FV home accessibility [53,60]	x		x	x
	Family barriers [61]	x		x	x
	Self efficacy [61]	x		x	x
	Child asking behaviors [53]	x		x	x
	Demographic data	x			
	Program appeal/use [26]			x	
	Newsletter click rate [26]		x		
**PROGRAM**	Process evaluation [49]	x	x	x	x

Note: * = Actigraph, LLC; Model GT3X+; Pensacola, FL.

### Hypotheses and statistical analyses

The primary hypothesis is that girls randomized to the treatment group will maintain a stable BMI percentile, while girls in the comparison and wait list control group will show an increase. Secondary hypotheses will examine group differences in FV, water, PA, and psychosocial variables over time. A repeated measures analysis of covariance will be conducted to examine intervention effects at post 1 and post 2, covarying baseline values. All analyses will control for potential confounding variables (e.g., family demographic characteristics and social desirability).

#### Anticipated results

At three months post intervention, girls in the treatment group will maintain a stable BMI percentile, while girls in the comparison and wait-list control groups will have an increase. Additionally, girls in the treatment group will have higher FV and water consumption, more minutes of moderate to vigorous PA, and fewer minutes of sedentary activity than girls in the comparison or wait-list control groups immediately after the intervention and 3 months post intervention. It is also expected that home availability of FV and PA equipment will mediate intervention effects on BMI percentile, FV consumption, and moderate to vigorous PA. Based on results of a small pilot study (n = 16) conducted prior to the outcome evaluation, we also anticipate high participation rates for both the online program and data collection activities, low attrition, and high compliance with setting, reporting, and achieving goals.

## Discussion/Conclusion

Online programs encouraging young African American girls to develop healthy diet and PA behaviors prior to adolescence are needed to reduce the risk of obesity and related diseases later in life. Procedures that contribute to behavioral change also need to be identified. Programs for ethnic minorities, however, must be culturally and developmentally appropriate and acceptable to key stakeholders, such as girls, parents, and the larger community to ensure community acceptance. They must also meet expectations of today’s savvy technology user in order to be adopted and used.

The behavioral program described in this paper presents the protocol guiding the development of a culturally and developmentally appropriate obesity prevention program for 8–10 year old African American girls. This research will provide important insights regarding the efficacy of an animated, online program presented in a comic book format at changing behavior, and it will also contribute to our understanding of behavioral components that facilitate change in this age group.

## Abbreviations

BMI: Body mass index; CNRC: Children’s Nutrition Research Center; F: Fruit; Fl oz: Fluid ounces; GEMS: Girls’ health Enrichment Multi-site Studies; PA: Physical activity; V: Vegetable.

## Competing interests

The authors declare that they have no competing interest.

## Authors’ contributions

DT is principal investigator of the study, conceived this project; guided the design of the intervention components; and drafted the paper. RM assisted with development and took the lead in beta testing. RBhatt assisted with design and managed the project during development. CB and DC assisted with design. IV wrote the procedures for the dietary data collection. CC wrote the recruitment and enrollment procedures. KC was a co-investigator and assisted with design. TB was the principal investigator of the original study; as a co-investigator, he assisted with design of the current study. YL is the study biostatistician and assisted with sample size determination and analysis plan. CW created the storyline. RBuday was the executive producer for the online program and guided software development, creation of sound tracks, and programming. All authors read and approved the manuscript.

## Authors’ information

RBhatt was with Department of Pediatrics, Baylor College of Medicine, when this program was developed; she is now with the Department of Family and Community Medicine, Baylor College of Medicine. CW is the playwright who wrote the story for the project. RBuday is the President of Archimage, Inc. (Houston, TX) and served as the program's executive producer.

## Pre-publication history

The pre-publication history for this paper can be accessed here:

http://www.biomedcentral.com/1471-2458/13/709/prepub
